# The HIV oligonucleotide database (HIVoligoDB)

**DOI:** 10.1093/database/bax005

**Published:** 2017-03-18

**Authors:** João Carneiro, Adriana Resende, Filipe Pereira

**Affiliations:** a Interdisciplinary Centre of Marine and Environmental Research (CIIMAR), University of Porto, Terminal de Cruzeiros do Porto de LeixAv. General Norton de Matos s/n 4450-208 Porto

## Abstract

The human immunodeficiency virus (HIV) is associated with one of the most widespread infectious disease, the acquired immunodeficiency syndrome (AIDS). The development of antiretroviral drugs and methods for virus detection requires a comprehensive analysis of the HIV genomic diversity, particularly in the binding sites of oligonucleotides. Here, we describe a versatile online database (HIVoligoDB) with oligonucleotides selected for the diagnosis of HIV and treatment of AIDS. Currently, the database provides an interface for visualization, analysis and download of 380 HIV-1 and 65 HIV-2 oligonucleotides annotated according to curated reference genomes. The database also allows the selection of the most conserved HIV genomic regions for the development of molecular diagnostic assays and sequence-based candidate therapeutics.

**Database URL:** http://portugene.com/HIVoligoDB

## Introduction

The human immunodeficiency virus (HIV) is a significant threat to public health, being associated with the acquired immunodeficiency syndrome (AIDS), one of the most widespread infectious diseases at global scale (1). Two HIV types have been identified. HIV-1 is responsible for most cases of AIDS while HIV-2 is confined mainly to West Africa, although several HIV-2 infections can be found in Mozambique, Angola, France and Portugal (2). HIV-2 infection are less virulent and transmissible than HIV-1, although HIV-2 is known to also cause AIDS (3). The HIV genome contains three major genes encoding essential enzymes and structural proteins: *gag*, *pol* and *env*. The single-stranded RNA genome is converted by the reverse transcriptase into a double-stranded DNA that is integrated into the genome of infected individuals (4). The process of genome replication is a source of genetic variability due to the absence of proofreading ability of the reverse transcriptase, resulting in a high mutation rate (5, 6).

A considerable number of nucleic acid techniques (NATs) have been developed for clinical diagnosis, genetic characterization of isolates, identification of genotypes, determination of viral loads, detection of drug resistance mutations and epidemiological studies (7, 8). In recent years, synthetic oligonucleotides complementary to HIV-1 RNA have been tested as possible antiviral or virucidal agents. Oligonucleotide-based therapeutics include antisense RNA, ribozymes, decoy RNAs, aptamers and small interfering RNA (9). Despite the abundance of DNA or RNA molecules targeting HIV, no organized repository of sequences is available. Some databases include genomic information for HIV-1 and HIV-2 (see online supplementary material for Figure S1). For example, the NCBI Probe Database (https://www.ncbi.nlm.nih.gov/probe) includes oligonucleotides for HIV, but lack any data on sequence conservation. The Los Alamos National Laboratory (http://www.hiv.lanl.gov/content/index) and the Stanford University (http://hivdb.stanford.edu/) HIV databases include information on genomic sequences and drug resistance mutations, but no information on oligonucleotides (10).

**Figure 1:**
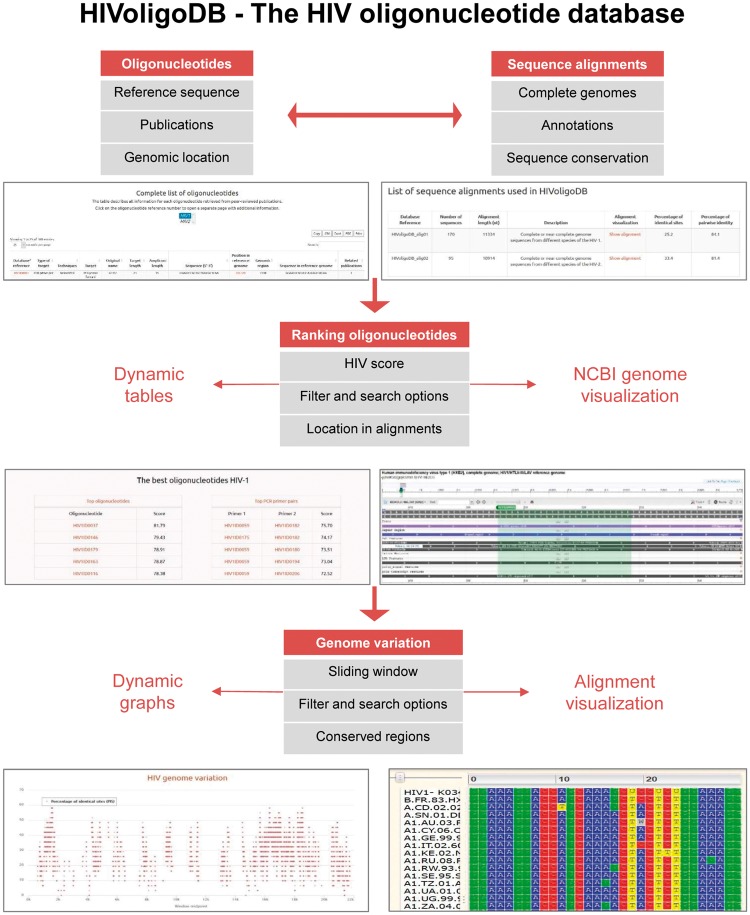
Screenshot of the data and tools included in the HIVoligoID.

Here, we present the HIV oligonucleotide database (HIVoligoDB) describing a set of oligonucleotides and genomic regions that can be used to improve the efficiency of HIV epidemiological studies and nucleic acid-based assays (Figure 1).

## Materials and Methods

The HIVoligoDB (http://portugene.com/HIVoligoDB) includes 380 HIV-1 (236 primers and 144 probes) and 65 HIV-2 oligonucleotides (57 primers and 8 probes) retrieved from 54 peer-reviewed publications and the NCBI Probe Database. Each oligonucleotide has a specific database code (for example, HIV1ID0001) and is associated with a reference genome (K03455.1 for HIV-1 and M15390 for HIV-2). The database provides descriptive webpages (e.g. type of target, related publications) for each oligonucleotide and a search engine to access dynamic tables with numeric data and multiple sequence alignments with complete HIV genomes. The multiple sequence alignments were retrieved from the Los Alamos National Laboratory (http://www.hiv.lanl.gov/content/index). The HIV-1 alignment includes sequences from all HIV-1 described sub-types (including non-M groups) (11). The HIV-2 alignment includes a carefully chosen subset of HIV-2 and SIV/SMM (simian immunodeficiency virus) sequences from the HIV Sequence Compendium (12).

The database ranks all oligonucleotides considering three main measures of sequence conservation: **(**a) percentage of identical sites (PIS), calculated by dividing the number of equal positions in the alignment for an oligonucleotide by its length; (b) percentage of identical sites in the last five nucleotides at the 3′ end of the oligonucleotide (3′PIS)—the most critical regions for an efficient binding to the template DNA during PCR and (c) percentage of pairwise identity (PPI), calculated by counting the average number of pairwise matches across the positions of the alignment, divided by the total number of pairwise comparisons. The ranking score (‘HIV score’) considers the mean value of the three different measures (PIS, 3′PIS and PPI). In theory, high PIS and PPI values indicates that an oligonucleotide binds to a conserved genomic region, increasing the probability of successful binding in a PCR or other technique. Calculations of melting temperature (*T*_m_) of each PCR primer were determined using the BioPython Melting Temperature module with Santa Lucia parameters (13) and the dimer free energy with the method used in Vienna Package (14). The melting temperatures and self-dimmer free energies are described in the table **‘**Oligonucleotides properties’ that are available through the menu **‘**Search’.

The database works with major web browsers (e.g. Internet Explorer, Mozilla Firefox, Chrome). The SQLite local database is used for data storage and runs on an Apache web server. The dynamic HTML pages were implemented using CGI-Perl and JavaScript and the dataset tables using the JQuery plugin DataTables v1.9.4 (http://datatables.net/). The database uses java graphics and dynamic tables. Python and perl algorithms implemented with BioPerl were used to perform identity and pairwise calculations.

## Results

The HIVoligoDB is a free resource with detailed records of oligonucleotides to study HIV-1 and HIV-2. The database has a core integration of multiple data from different sources, which facilitates the analysis of hundreds of records through search and filtering processes. The HIVoligoDB is being constantly updated with new oligonucleotides added once they are described in peer-reviewed publications. We have used the HIVoligoDB to perform calculations over 445 oligonucleotides located in different HIV-1 and HIV-2 genomic regions (see online supplementary material for Figure S2). The multiple sequence alignment of HIV-1 (alig01) has a PIS of 15.26% and a PPI of 85.46%. The HIV-2 alignment (alig02) has a PIS of 29.09% and a PPI of 83.20%. The researcher can use the database to access the most conserved oligonucleotides (Table 1). Our analyses revealed that oligonucleotides HIV1ID0037 (HIV score of 81.79%), HIV1ID0146 (79.43%) and HIV1ID0179 (78.91%) are the most conserved for HIV-1. The HIV-1 PCR primer pair with the highest HIV score (80.35%) was HIV1ID0037–HIV1ID0179. The most conserved oligonucleotides for HIV-2 were HIV2ID0015 (95.14%), HIV2ID0061 (87.49%) and HIV2ID0057 (84.71%). The PCR primer pair with the highest HIV score (91.31%) for HIV-2 was HIV2ID0015–HIV2ID0061. Similar melting temperatures (*T*_m_) in oligonucleotide pairs are important for the efficiency of PCR (15). We identified some PCR primer pairs with similar *T*_m_ and high HIV scores. For example, the primers HIV1ID0037 (*T*_m_ of 55.97 °C) and HIV1ID0179 (*T*_m_ of 58.72 °C) have similar *T*_m_ values and a high HIV score (80.35).

**Table 1:** Oligonucleotides with highest HIV score for HIV-1 and HIV-2

Virus	Code	Type	Original name	Target length	Sequence (5′–3′)	Position in reference genome	Genomic region	HIV Score
HIV-1	HIV1ID0037	PCR primer reverse	NP174	21	CTACYGCCCCTTYACCTTTCC	4957-4977	*pol*	81.79
	HIV1ID0146	Degenerate PCR primer	env27F	19	CTGGYATAGTGCARCARCA	7861-7879	*env*	79.43
	HIV1ID0179	PCR primer forward	HPOL4235	23	CCCTACAATCCCCAAAGTCAAGG	4653-4675	*pol*	78.91
	HIV1ID0163	PCR primer forward	intF	20	CCCTACAATCCCCAAAGTCA	4653-4672	*pol*	78.87
	HIV1ID0116	PCR primer forward	E180	20	GTCTGGTATAGTGCAACAGCA	7860-7879	*env*	78.38
	HIV1ID0182	PCR primer reverse	HPOL4481	21	GCTGTCCCTGTAATAAACCCG	4899-4919	*pol*	77.9
	HIV1ID0283	siRNA antisense	si4652	21	UGACUUUGGGGAUUGUAGGGA	4652-4672	*pol*	
	HIV1ID0017	Probe	H-1 probe	23	AATGAGGAGGCTGCAGAATGGGA	1405-1427	*gag*	77.24
	HIV1ID0087	PCR primer forward	G80	23	ATGAGAGAACCAAGGGGAAGTGA	1471-1493	*gag*	76.61
	HIV1ID0052	PCR primer reverse	gag R4	32	TTGATGGTCTCTTTTAACATTTGCATGGCTGC	1375-1406	*gag*	76.18
HIV-2	HIV2ID0015	PCR primer reverse	A-Loop B	21	AATTTTAAAAGAAGGGGAGGA	4607-4627	*pol*	95.14
	HIV2ID0061	PCR primer forward	POL OG 479 INV	23	GGAGCAGTCCTAGTCAAGGTAGG	4796-4818	*pol*	87.49
	HIV2ID0057	PCR primer reverse	PFD INV	21	CTGCCTTCTCTGAAATAGACC	4729-4749	*pol*	84.71
	HIV2ID0044	PCR primer forward	EB7	17	CCYAGGCAAGCATGGTG	7149-7165	*env*	83.47
	HIV2ID0017	PCR primer forward	Seq.ID.NO.19	26	CCTCAATTCTCTCTTTGGAAAAGACC	2084-2109	*gag*	82.91
	HIV2ID0041	PCR primer reverse	EB6	20	CCATTRAAGCCAAACCAWGT	6945-6964	*env*	81.32
	HIV2ID0060	PCR primer reverse	POL OG 479	23	CCTACCTTGACTAGGACTGCTCC	4796-4818	*pol*	80.82
	HIV2ID0043	PCR primer reverse	EB5	20	CTCCTCTGCAGTTAGTCCAC	7310-7329	*env*	80.61
	HIV2ID0003	PCR primer forward	SR64, envF	23	CTCCAGGCAAGAGTCACTGCTAT	7869-7891	*env*	80.44
	HIV2ID0055	PCR primer forward	X410	21	CACCTCAATTCTCTCTTTGGA	2082-2102	*gag*	80.09

Extensive genetic diversity has been observed in HIV (16), and several works have used a multiplicity of techniques to study these viruses (16–23). The HIV score allows users to select the most accurate oligonucleotides for different techniques. The oligonucleotides can also be associated with the technique where they were used by the first time. Moreover, each oligonucleotide can be easily associated with the respective region in the reference genome.

The most conserved regions for HIV-1 were the *gag* and *pol*, while LTRs had the lowest HIV scores (see online supplementary material for Figure S3). The protease and p51 RT regions (located inside *pol* region) include 11% (43 oligonucleotides) of all oligonucleotides. The PCR primers’ pairs for HIV-1 with the highest HIV score in these genomic regions were HIV1ID0073–HIV1ID0206 (score of 73.61 and *T*_m_ of 58.9 °C and 60.9 °C) and HIV1ID0035–HIV1ID087 (a score of 73.28, *T*_m_ of 53.9 °C and 57.1 °C). For HIV-2, the oligonucleotides with highest conservation scores were those located in the *pol* region, and the lowest located in the *tat* region (see online supplementary material for Figure 3B). The *pol* region included 21.5% of all oligonucleotides. The primers pairs for HIV-2 with the highest HIV score located in the *pol* region were HIV2ID0015HIV2ID0061 (a score of 91.31, *T*_m_ of 49.40°C and 58.80°C) and HIV2ID0057- HIV2ID0061 (a score of 86.1, *T*_m_ of 51.65°C and 58.80°C). By combining different set of PCR primers, a complete amplification of the protease and p51 RT regions can be performed for detection of drug resistant mutations.

We calculated the PIS and PPI values for all 30 nt sliding windows across the HIV-1 and HIV-2 alignments, which allow the identification of the most conserved genomic regions in these viruses (see online supplementary material for Figures S4 and S5). This information can be used to design new oligonucleotides.

## Examples of Use

In order to select the oligonucleotides located in the most conserved HIV-1 genomic regions, the user can go to the ‘Search’ tab on the top menu bar, and open the ‘The best oligonucleotides’ tab. The table is automatically ordered by the ‘HIV Score’ column filter. The user can now access the oligonucleotide information by clicking in the oligonucleotide ID hyperlink, which shows the summary information for that record. The database can also be used to filter all columns using the search tool. For instance, to access the best oligonucleotide for HIV-1 located in *pol* region, the user can type ‘pol’ in the search box. The database table now shows only the records where the word pol is found. The oligonucleotide with highest ‘HIV score’ in the *pol* region is HIV1ID0037.

If the aim is to design a new oligonucleotide, the user can click on the ‘Genome variation’ tab on the top menu bar. The user can then select the alignment to display in the graph (HIV-1 or HIV-2), which describe a sliding window analysis of 30 nt windows with 1 nt of overlap of PIS and PPI values. The list of the most conserved genomic regions can be found in a table. For example, the genomic region 5524–5554 of the HIV-1 alignment has the highest PIS value (64.52%). This section of the alignment can be visualized by clicking on the 5524–5554 value in the table. The user can also visualize any window of the alignment by using the ‘Show window in alignment’ box.

## Funding

Portuguese Foundation for Science and Technology (FCT), European Regional Development Fund (ERDF) [IF/01356/2012 to F.P. and SFRH/BPD/100912/2014 to J.C.] and Direção-Geral da Saúde and Coordenação Nacional para a Infeção VIH/SIDA [VIH/SAU/0011/2011]. CIIMAR—FCT and ERDF [UID/Multi/04423/2013].

## Supplementary data

Supplementary data are available at *Database* Online.

***Conflict of interest***: None declared.

## Supplementary Material

Supplementary Data
