# Supplementary material for: The HIV oligonucleotide database (HIVoligoDB)
Source: Database (Oxford). 2017 Mar 18;2017:bax005. doi: 10.1093/database/bax005 (PMC5502365; doi:10.1093/database/bax005)
Supplement: Supplementary Data [file bax005_Supp.pdf]

Supplementary data

The HIV oligonucleotide database (HIVoligoDB)

João Carneiro, Adriana Resende and Filipe Pereira

Interdisciplinary Centre of Marine and Environmental Research (CIIMAR), University of Porto, Porto, Portugal.

Supplementary Figure S1: Description of the main features available in the HIVoligoDB and other HIV databases.

Database	*Sequence search*	*Geographic search*	*Mutation search*	*Genome display*	*Oligonucleotides*	*Ranking tables*	*Genome diversity*	*Genotype Correlations*	*Gene expression profile*	*Clinical data*
**HIVoligoDB**	Yes	No	No	Yes	Yes	Yes	Yes	No	No	No
**HIV Los Alamos databases**	Yes	Yes	No	Yes	No	No	No	No	No	No
**HIV drug resistance database**	No	No	Yes	No	No	No	Yes	Yes	No	Yes
**HIV-1 Human Interaction Database**	Yes	No	No	Yes	No	No	Yes	No	No	No
**Virus mentha**	No	No	No	No	No	No	No	No	No	No
**GuavaH**	No	No	No	No	No	No	Yes	No	Yes	Yes
**HIV/AIDS Surveillance Data Base**	No	Yes	No	No	No	No	No	No	No	Yes

Supplementary Figure S2: Localization of oligonucleotides (arrows) in the HIV-1 (A) and HIV-2 (B) reference genomes.

A

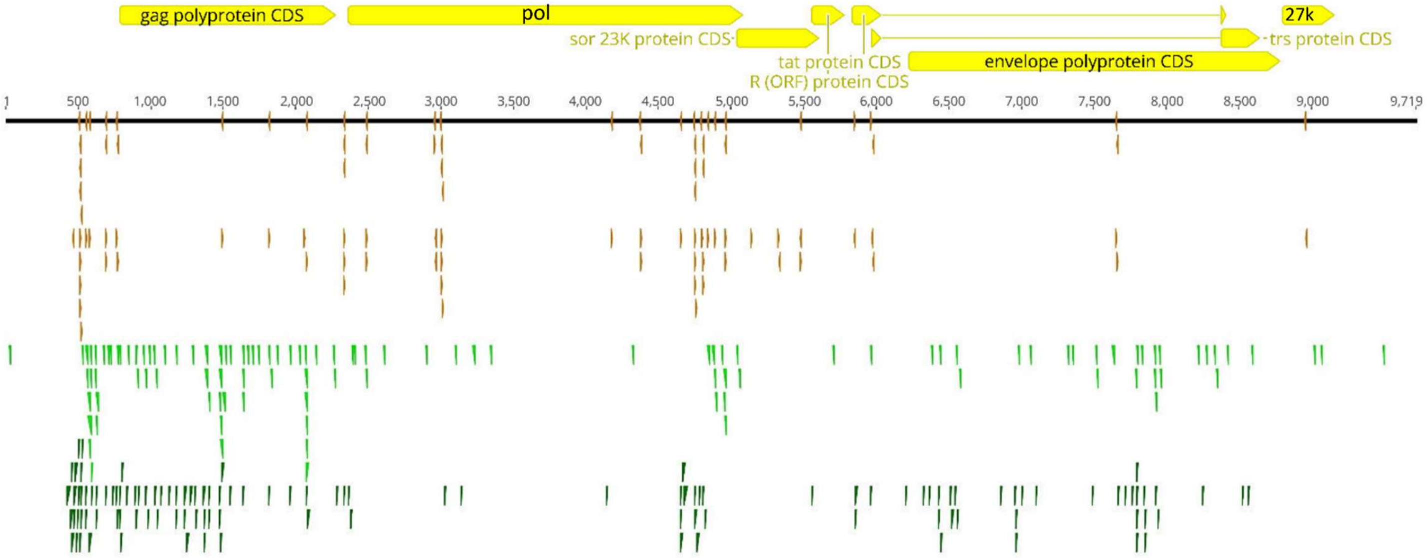

B

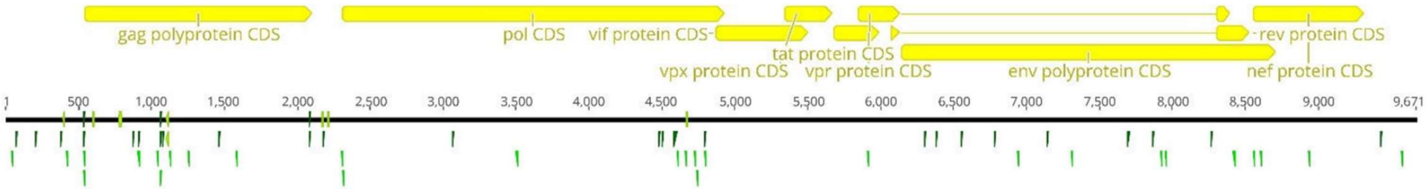

Supplementary Figure S3: HIV scores estimated in each HIV-1 and HIV-2 genomic region. The mean is represented by Xs and outliers are represented by dots.

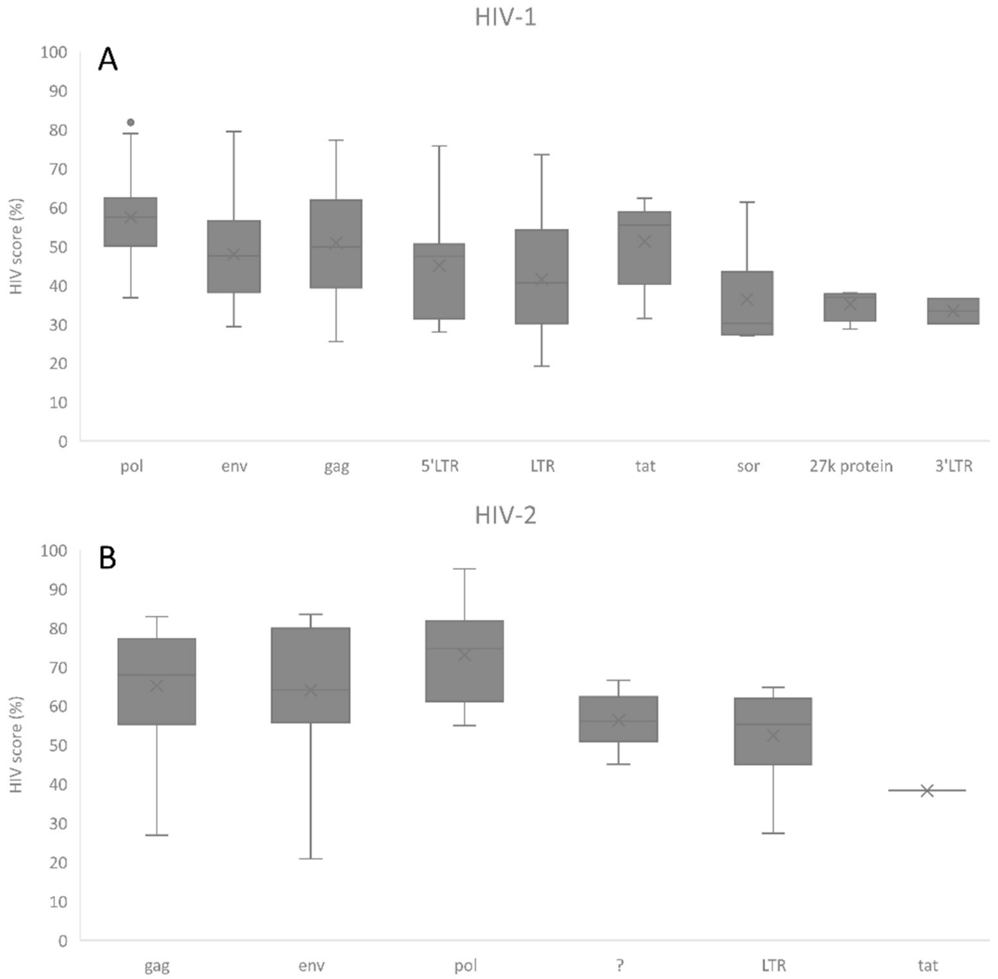

Supplementary Figure S4: Genetic diversity across the HIV-1 and HIV-2 genomes. The plots display the percentage of identical sites (PIS) and percentage of pairwise identity (PPI) in a sliding window analysis (30 nt windows with 1 nt of overlap) estimated in multiple sequence alignments with complete or near complete genome sequences of HIV-1 and HIV-2.

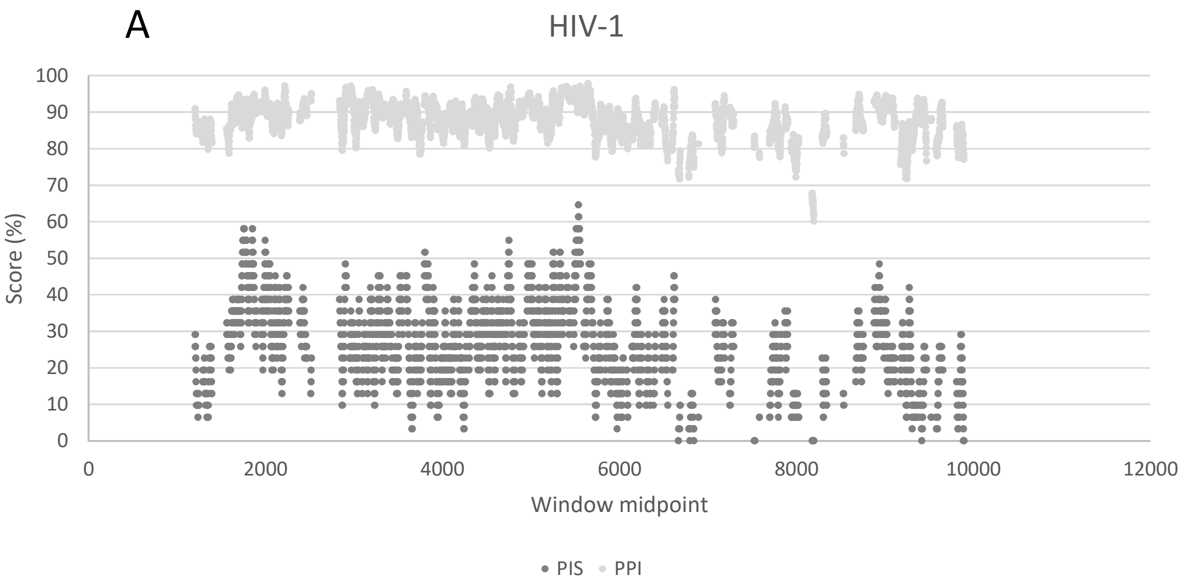

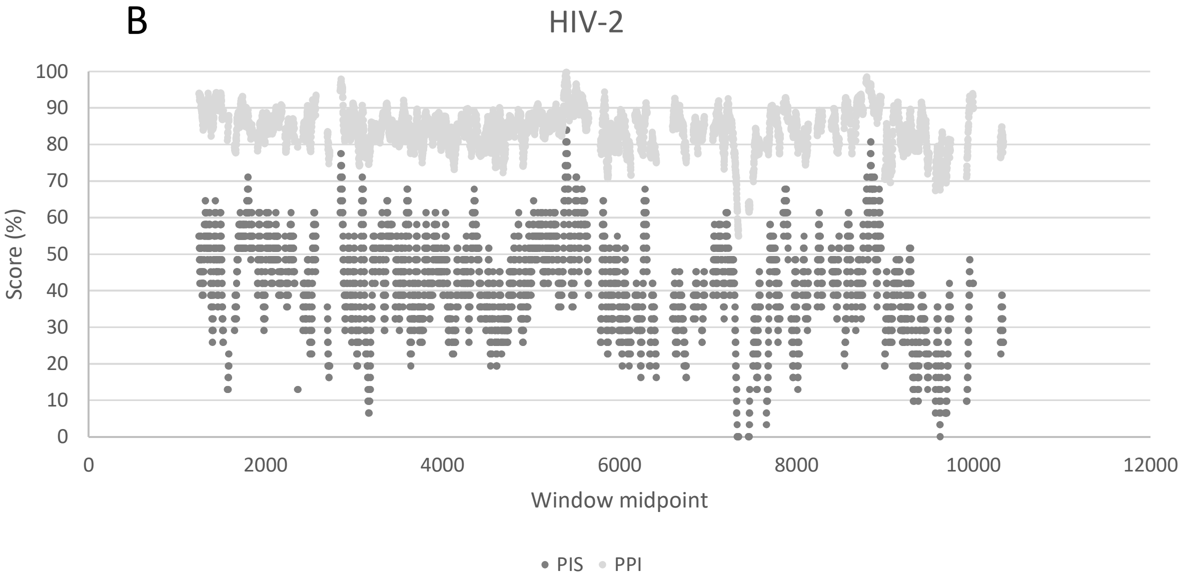

Supplementary Figure S5: HIV-1 genomic regions determined with the sliding window method (30 nucleotides with step of 1 nucleotide) that present the highest percentage of identical sites (PIS) and percentage of pairwise identity (PPI) values.

**Genome Position**	**PIS (%)**
**5524-5554**	64.52
**5523-5553**	61.29
**5525-5555**	61.29
**5526-5556**	61.29
**5527-5557**	61.29
**5529-5559**	61.29
**5530-5560**	61.29
**1738-1768**	58.06
**1739-1769**	58.06
**1741-1771**	58.06
	PPI (%)
**5631-5661**	97.72
**5636-5666**	97.72
**5632-5662**	97.68
**5634-5664**	97.68
**5635-5665**	97.68
**5633-5663**	97.65
**2204-2234**	97.18
**2953-2983**	97.1
**2952-2982**	97.06
**5544-5574**	97.01
